# The hsa-miR-181a-5p reduces oxidation resistance by controlling SECISBP2 in osteoarthritis

**DOI:** 10.1186/s12891-018-2273-6

**Published:** 2018-10-05

**Authors:** Jianli Xue, Zixin Min, Zhuqing Xia, Bin Cheng, Binshang Lan, Fujun Zhang, Yan Han, Kunzheng Wang, Jian Sun

**Affiliations:** 1 Department of Orthopaedics, The Second Affiliated Hospital, Xi’an Jiaotong University Health Science Center, 157 West 5th Road, Xi’an, Shaanxi 710004 People’s Republic of China; 2 Department of Biochemistry and Molecular Biology, School of Basic Medical Sciences, Xi’an Jiaotong University Health Science Center, Xi’an, Shaanxi 710061 People’s Republic of China; 3 Beaurau of healthcare, Shaanxi Health and Family Planning Commission, Xi’an, Shaanxi 710000 People’s Republic of China

**Keywords:** *miRNA-181a-5p*, SECISBP2, Selenoprotein, Cartilage, Osteoarthritis

## Abstract

**Background:**

The phenotypes of osteoarthritis (OA) consist of cartilage extracellular matrix (ECM) metabolism disorder and the breakdown of cartilage homeostasis, which are induced by pro-inflammatory factors and oxidative stress. Selenoproteins regulated by selenocysteine insertion sequence binding protein 2 (SBP2) are highly effective antioxidants, but their regulatory mechanisms, particularly the involvement of miRNAs, are not fully understood.

**Methods:**

To explore whether *miR-181a-5p* and *SBP2* are involved in OA pathogenesis, we established an IL-1β model using the chondrocyte SW1353 cell line. Next, we up- or down-regulated *SBP2* and *miRNA-181a-5p* expression in the cells. Finally, we measured the expression of *miRNA-181a-5p*, *SBP2* and three selenoproteins in OA cartilage and peripheral blood.

**Results:**

The results showed that IL-1β increased *hsa-miR-181a-5p* and decreased *SBP2* in a time- and dose-dependent manner. *GPX1* and *GPX4*, which encode crucial glutathione peroxidase antioxidant enzymes, were up-regulated along with *SBP2* and *miR-181a-5p*. Furthermore, *SBP2* showed a significant negative correlation with *miR-181a-5p* during induced ATDC5 cell differentiation. There was lower *GPX1* and *GPX4* mRNA expression and SBP2 protein expression in damaged cartilage than in smooth cartilage from the same OA sample, and *hsa-miR-181a-5p* expression on the contrary. Similar results were observed in peripheral blood. In conclusion, we have reported a novel pathway in which pro-inflammatory factors, miRNA, SBP2 and selenoproteins are associated with oxidation resistance in cartilage.

**Conclusion:**

Overall, this study provides the first comprehensive evidence that pro-inflammatory factors cause changes in the cartilage antioxidant network and describes the discovery of novel mediators of cartilage oxidative stress and OA pathophysiology. Our data suggest that *miR-181a-5p* may be used to develop novel early-stage diagnostic and therapeutic strategies for OA.

**Electronic supplementary material:**

The online version of this article (10.1186/s12891-018-2273-6) contains supplementary material, which is available to authorized users.

## Background

Osteoarthritis (OA) may be a response to superfluous mechanical stress or inflammation, and pro-inflammatory factors, including interleukin-1 (IL-1β), interleukin-6 (IL-6), and tumour necrosis factor-α (TNF-α), are involved in OA pathogenesis [1, 2]. The phenotypes of cartilage injury processes induced by pro-inflammatory factors are cartilage extracellular matrix (ECM) metabolic disorder, the disruption of cartilage homeostasis, and enhanced expression of matrix degradation enzymes such as MMP13 [3]. MMP13, a major enzyme hydrolysing type-II collagen (COL2), is a dominant protein component of the cartilage ECM [4, 5] and a biomarker for arthritis progression and therapeutic effects [6–8].

Reactive oxygen species (ROS) are products of aerobic metabolism that injure DNA, proteins, and cellular membranes [9–11]. Oxidative stress plays important roles in the pathogenesis of OA and cartilage degradation, which is induced by ROS, and traumatic loading increases cartilage oxidation and causes cell death [12]. In addition, oxidative stress-mediated regulation of the expression of redox-sensitive proteins is regarded as a key mechanism underlying age-related cellular dysfunction and disease progression [13].

Selenoproteins (Sel) are important members of a network of antioxidant enzymatic systems and minimize damage induced by ROS. They contain selenocysteine (Sec), the 21st proteinogenic amino acid, which is named after the essential biological trace element selenium (Se) and acts as an active-site residue essential for the catalytic activity of selenoproteins [9–11]. The genetic code ‘UGA’, commonly a termination codon in cells, encodes Sec into selenoproteins [14]. Several special cis-trans elements and trans-acting factors, typically the Sec insertion sequence (SECIS) and Sec insertion sequence binding protein 2 (SECISBP2 or SBP2), regulate selenoprotein biosynthesis [15, 16]. SECIS, which is located in the selenoprotein mRNA 3′-untranslated region (3′-UTR), binds with SBP2. The function of SBP2 is to carry Sec-tRNA^Sec^ into the ribosome ‘A site’ to recognize ‘UGA’ as the Sec codon during selenoprotein synthesis [15, 16].

Intriguingly, osteo-chondroprogenitor-specific deletion of the selenocysteinyl tRNA^Sec^ gene results in dyschondroplasia phenotypes, particularly those showing abnormal skeletal development in mice [17]. ‘UGA’ is recognized as a termination codon, and inactive truncated selenoproteins are produced in the presence of insufficient Sec-tRNA^Sec^ [18]. Similarly, the TrxR1 short inactive fragment, a two-amino-acid truncated C-terminal motif, leads to the death of human lung carcinoma A549 cells [19]. However, little is known about how selenoprotein biosynthesis regulates OA cartilage. In particular, the pathway from pro-inflammatory factors to selenoprotein biosynthesis mediated by SBP2 in cartilage is poorly understood.

Moreover, more than 20 miRNAs, such as the cartilage-specific *miR-140-5p*, participate in chondrogenesis, cartilage homeostasis and degradation, and chondrocyte metabolism, which are closely associated with OA development [20–22]. Further, miR-9, miR-34a and miR-146a are related with oxidative stress in OA chondrocytes [23, 24]. In a previous study, we identified a repertoire of miRNAs during the development of rat femoral articular cartilage [25] and demonstrated that *miR-337* regulates chondrogenesis through a direct target, TGFBR2 [26]. Specifically, *miR-181a-5p*, a member of the *miR-181* family, which is organized into three clusters (*miR-181a/b-1*, *miR-181a/b-2*, and *miR-181c/d*), is positively correlated with chondrogenesis [25]. Meanwhile, non-hypertrophic articular and hypertrophic MSC-derived chondrocytes showed differential expression of *miR-181a-5p*, suggesting that its expression is altered during successive differentiation stages [27]. Moreover, *miR-181a-5p* is predicted to be a target of *hSBP2* by TargetScanHuman7.1, and it may inhibit the expression of the important ECM protein aggrecan (ACAN) in chondrocytes, simultaneously acquiring a negative feedback function for cartilage homeostasis [28]. However, further investigation is required to understand the oxidation resistance-associated roles of *miR-181a-5p* in OA.

In the present study, the glutathione peroxidase-encoding genes *GPX1* and *GPX4* and the selenoprotein S-encoding gene *SELS* were examined due to their regulation by *SBP2*. Hence, we investigated the detailed regulatory relationships among pro-inflammatory factors, miRNA, SBP2 and selenoproteins in the context of oxidation resistance in cartilage. Overall, this study provides the first comprehensive evidence for changes in pro-inflammatory factors in the cartilage antioxidant network during OA and describes the discovery of novel mediators of cartilage oxidative stress and OA pathophysiology. Therefore, our data suggest that *miR-181a-5p* may be useful for the development of novel early-stage diagnostic and therapeutic strategies for OA.

## Methods

### Cell culture

The human chondrosarcoma chondrocyte SW1353 cell line was obtained from the Chinese Academy of Sciences (Shanghai, China) and cultured in RPMI-1640 medium (HyClone, USA) with 10% foetal bovine serum (ExCell, China). The murine chondroblast ATDC5 cell line was obtained from the European Collection of Cell Cultures (ECACC) and maintained in Dulbecco’s Modified Eagle’s medium/Ham’s F12 medium (DMEM/F12, HyClone, USA) supplemented with 5% FBS (Gibco, USA). Both cell lines were maintained in a humidified incubator with 5% CO_2_ at 37 °C, cultured in monolayers and grown to confluence. The medium contained 1% penicillin/streptomycin (Sigma, USA). The cells were seeded in 12-multiwell plates at 7 × 10^4^ cells/well.

For the cartilage matrix degradation model, SW1353 cells were placed in FBS-free medium for more than 10 h, and then the cells were incubated with 0 (as control), 1, 5, 10 and 20 ng/ml IL-1β (Sino Biological Inc., China) for 12 h, or 10 ng/ml IL-1β for 0 (as control), 6, 12, 24 and 48 h. For the chondrocyte differentiation model, ATDC5 cells were induced with 1× ITS supplement (1 mg/ml insulin, 0.55 mg/ml transferrin and 0.5 μg/ml selenium) added to the medium. The chondrogenic culture medium was changed every day.

### Transient transfection of hsa-miR-181a-5p mimics or inhibitor sequences

SW1353 cells were seeded for 24 h, and 50 nM *hsa-miR-181a-5p* mimics (*mimic-181a-5p*) or negative control (*mimic-NC*) (Genepharma, China) and 200 nM *hsa-miR-181a-5p* inhibitor (*inhibitor-181a-5p*) or negative control (*inhibitor-NC*) (Genepharma, China) were transiently transfected into SW1353 cells by 1.5 μl/well Lipofectamine™ 2000 (Invitrogen, USA) according to the manufacturer’s instructions. Information about *miR-181a-5p* is provided in Tables 1 and 2.

**Table 1:** Information of Mature *miR-181a-5p*

ID	Accession	Mature sequence (5′-3′)
*hsa-miR-181a-5p*	MIMAT0000256	AACAUUCAACGCUGUCGGUGAGU
*mmu-miR-181a-5p*	MIMAT0000210

**Table 2:** Information of Stem-loop *hsa-miR-181a*

ID	Accession	Location	Stem-loop sequence (5′-3′)
*hsa-mir-181a-1 (hsa-mir-213)*	MI0000289	1q32.1	UGAGUUUUGAGGUUGCUUCAGUGAACAUUCAACGCUGUCGGUGAGUUUGGAAUUAAAAUCAAAACCAUCGACCGUUGAUUGUACCCUAUGGCUAACCAUCAUCUACUCCA
*hsa-mir-181a-2*	MI0000269	9q33.3	AGAAGGGCUAUCAGGCCAGCCUUCAGAGGACUCCAAGGAACAUUCAACGCUGUCGGUGAGUUUGGGAUUUGAAAAAACCACUGACCGUUGACUGUACCUUGGGGUCCUUA

### Transient transfection of siRNAs and plasmids

The full-length human *SBP2* CDS was cloned from SW1353 chondrocyte cDNA and inserted into a *pEFGP-N1* vector (Invitrogen, USA). The primer sequences for the *hSBP2-CDS* clone are listed in Table 3. SW1353 cells were seeded for 24 h, and 1, 1.5, 2 and 4 μg of the *pEFGP-mSBP2-N1* vector or empty vector were transiently transfected into cells by 1.5 μl/well Lipofectamine™ 2000 (Invitrogen, USA). The expression of exogenous and endogenous SBP2 was determined by western blotting with an anti-SBP2 antibody after transfection for 24 h.

**Table 3:** Information of human primers for *hSBP2-CDS*

Gene	Sequence (5′-3′)
*hSBP2-CDS-Forward*	CAGGTCGGATCCAGA**CCCGGG**gccaccATGGCGTCGGAGGGG
*hSBP2-CDS-Reverse*	TCTGTAGAATTCGGT**CCCGGG**TAAATTCAAATTCATCAT

Additionally, *hSBP2* siRNA (*si-SBP2*) and control siRNA (*si-NC*) sequences were purchased from Genepharma Biotechnology Inc. (Genepharma, China). Cell transfection was performed according to the manufacturer’s instructions. For gene knockdown, SW1353 cells were seeded for 24 h, and 50 nM *si-SBP2* (5′-GAGCCACACUACAUUGAAATT-3′) or *si-NC* was transiently transfected into the cells by 1.5 μl/well Lipofectamine™ 2000 (Invitrogen, USA) according to the manufacturer’s instructions. Knockdown efficiency was determined by western blotting after transfection for 48 h.

### Patients and articular cartilage collection

OA patients were diagnosed according to the modified Outerbridge classification by The Second Affiliated Hospital, Xi’an Jiaotong University Health Science Center. Articular cartilage samples were obtained at the time of total knee replacement (TKR) from 10 human patients with knee OA (6 women and 4 men; mean ± SEM age: 60 ± 8.3 y) from Shaanxi province, China. All patients were diagnosed with Kellgren and Lawrence grade IV OA. After washing with sterile phosphate buffered saline (PBS), portions of cartilage with a damaged articular surface and portions with a smooth articular surface were used for RNA extraction and immunohistochemistry. Smooth cartilage samples were carefully assessed for any gross signs of degeneration or injury, and only normal-appearing smooth cartilage was used as an internal control (a self control). All cartilage samples were collected without fibrillation. Peripheral blood samples were obtained from 20 OA patients (14 women and 6 men; mean ± SD age: 66.6 ± 5.7 y) and 20 normal control patients (14 women and 6 men; mean ± SD age: 65.9 ± 3.1 y).

### Total RNA extraction and quantitative PCR analysis

For RNA extraction, cartilage tissues were harvested from smooth articular surfaces and damaged articular surfaces of the same patient and chopped into pieces that were smaller than 2 × 2 mm. Then, the pieces were immediately frozen in liquid nitrogen. Total RNA was isolated from cells, tissue pieces or plasma samples using TRIzol® (Invitrogen, USA). cDNA was synthesized from 2 μg of total RNA according to the manufacturer’s instructions (RevertAid™; Fermentas, Canada) in a final volume of 20 ml and stored at − 20 °C until use. Furthermore, miRNA-cDNA was obtained using the One Step PrimeScript® miRNA cDNA Synthesis Kit (Takara, Japan).

Both mRNA and miRNA expression was tested by 10 μl real-time quantitative PCR (RT-qPCR), which was performed on an iQ5 real-time PCR detection system (Bio-Rad, Hercules, CA, USA) with SYBR® Premix Ex Taq™ II (TaKaRa, Japan). Relative gene expression was normalized against *GAPDH* expression in SW1353 cells or *β-Actin* expression in ATDC5 cells. Additionally, let-7a was used as the internal reference for *miR-181a-5p*. The procedure for miRNA-cDNA qPCR consisted of two-step amplification: pre-denaturation at 95 °C for 10 s, followed by PCR amplification with 40 cycles of 95 °C for 5 s and 60 °C for 20 s. Information about the primers and PCR amplification is provided in Tables 4, 5 and 6.

**Table 4:** Information of miRNA-181a-5p for Real-time PCR

MicroRNAs	Accession NO.	Forward primer (5′-3′)
*hsa-miRNA-181a-5p*	MIMAT0000858	CGCAACATTCAACGCTGTC
*hsa-let-7a*	MIMAT0000774	CGCTGAGGTAGTAGGTTGT
Reverse primer: GTGCAGGGTCCGAGGT

**Table 5:** Information of mouse primers for Real-time PCR

Gene	Sequence (5′-3′)	Product size (bp)	Annealing temperature (°Χ)
*Sbp2*	Forward:CTGCTCCAAAGGCCAAAG	195	60
	Reverse:GTGATTGCCCTCTGTGTCTTC		
*β-Actin*	Forward:AACAGTCCGCCTAGAAGCAC	281	60
	Reverse:CGTTGACATCCGTAAAGACC

**Table 6:** Information of human primers for Real-time PCR

Gene	Sequence (5′-3′)	Product size (bp)	Annealing temperature (°C)
*SBP2*	Forward: CCGCAGATTCAGGGATTACT	92	60
	Reverse: CTTGGAAACGGACCAGTTCT		
*ACAN*	Forward: GGCATTTCAGCGGTTCCTTCTC	135	60
	Reverse: AGCAGTTGTCTCCTCTTCTACGG		
*MMP13*	Forward: AATATCTGAACTGGGTCTTCCAAAA	102	60
	Reverse: CAGACCTGGTTTCCTGAGAACAG		
*COL2A1*	Forward: TGGACGATCAGGCGAAACC	244	62
	Reverse: GCTGCGGATGCTCTCAATCT		
*GPx1*	Forward: AAGCTCATCACCTGGTCTCC	124	60
	Reverse: CGATGTCAATGGTCTGGAAG		
*GPx4*	Forward: GCTGTGGAAGTGGATGAAGA	105	60
	Reverse: TGAGGAACTGTGGAGAGACG		
*SELS*	Forward: CACCTATGGCTGGTACATCG	130	60
	Reverse: AACATCAGGTTCCACAGCAG		
*GAPDH*	Forward: CACCCACTCCTCCACCTTTG	110	64
	Reverse: CCACCACCCTGTTGCTGTAG		

### Protein sample preparation and western blotting

Total protein samples from SW1353 cells or ATDC5 cells (10–20 μg) were separated by 10% SDS-PAGE and transferred to PVDF membranes (EMD Millipore, Darmstadt, Germany). After blocking with 3% non-fat milk in TBST buffer, the membranes were incubated with primary antibodies followed by secondary antibodies conjugated to horseradish peroxidase (HRP) and visualized using an ECL detection system (EMD Millipore, Darmstadt, Germany) on a chemiluminescence imaging system. The primary antibodies included anti-SBP2 (1:500, CA, USA), anti-GPX1 (1:2000, CA, USA), anti-MMP13 (1:1000, Abcam, USA) and anti-β-ACTIN (1:2000, Proteintech, China). The following secondary antibodies were purchased from Beyotime Biotech (Jiangsu, China): horseradish peroxidase-coupled anti-rabbit (1:5000) and anti-mouse (1:5000).

### Immunohistochemistry staining

After measuring intrinsic peroxidase activity, articular cartilage sections were blocked with 3% hydrogen peroxide (H_2_O_2_) and then incubated with 1.5% BSA for 1 h. The sections were covered with anti-SBP2 antibodies (1:250, CA, USA) and incubated at 4 °C in a wet box. After 14 h, all sections were rinsed with PBS and then sequentially incubated with biotinylated secondary antibody for 1 h and DAB reagent (Boster, Wuhan, China) for 5 min at room temperature. Chromogenic reactions were terminated once claybank regions were observed under a microscope. Rabbit IgG was used as a negative control.

### Statistical analysis

Data are presented as the mean ± SEM. The statistical significance of pathological data was calculated by using the Mann-Whitney U test. Means of two groups were compared using Student’s t test, and statistical significance was achieved at *P* < 0.05 in all tests (*: *P* < 0.05, **: *P* < 0.01 and **: *P* < 0.001). All analyses were performed using GraphPad Prism 6.0 (GraphPad Software, San Diego, CA, USA).

## Results

### Both hsa-miR-181a-5p and SBP2 are regulated by IL-1β in chondrocytes

**Fig. 1:**
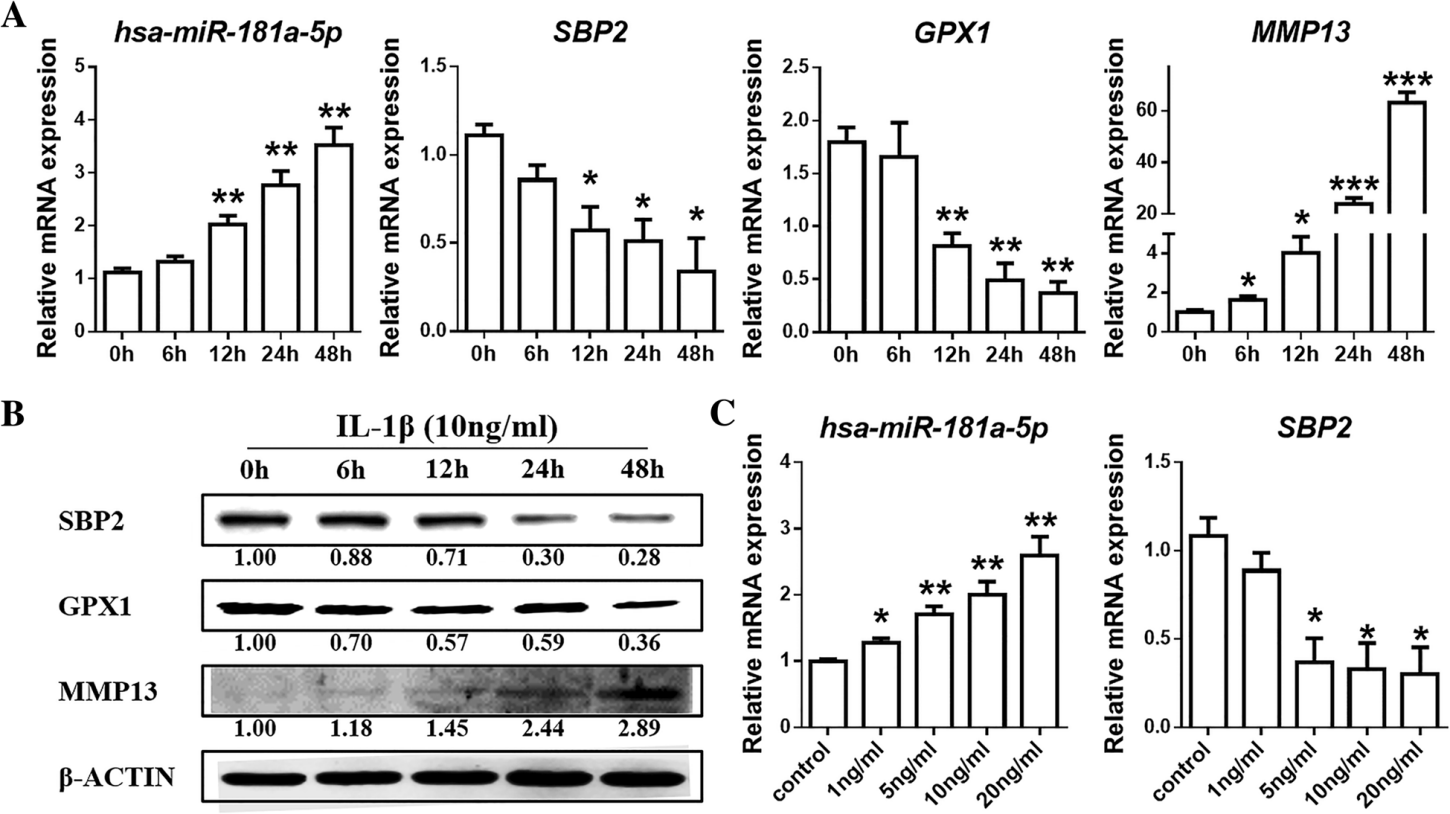
Both hsa-miR-181a-5p and SBP2 are regulated by IL-1β in chondrocytes. **a** The expression of hsa-miRNA-181a-5p, SBP2, GPX1 and MMP13 after treatment with 10 ng/ml IL-1β for 0 (as control), 6, 12, 24 and 48 h in SW1353 cells. (*n* = 3, 3). **b** The expression of SBP2, GPX1 and MMP13 after treatment with 10 ng/ml IL-1β for 0 (as control), 6, 12, 24 and 48 h in SW1353 cells. **c** The expression of hsa-miRNA-181a-5p and SBP2 after treatment with 0 (as control), 1, 5, 10 and 20 ng/ml IL-1β for 12 h in SW1353 cells. (*n* = 3, 3). The data are expressed as the mean ± SEM; *, ** and *** indicate *P* < 0.05, 0.01 and 0.001, respectively

IL-1β was selected to stimulate SW1353 cells, and *hsa-miR-181a-5p* expression levels were determined by stem loop RT-qPCR. The expression of *hsa-miR-181a-5p* and *MMP13* continuously and robustly increased after treatment with 10 ng/ml IL-1β for 0 (as a control), 6, 12, 24 and 48 h in SW1353 cells, while *SBP2* and *GPX1* expression was continuously and sharply reduced at the mRNA level (Fig. 1a). Meanwhile, SBP2, GPX1 and MMP13 expression at the protein level showed the same patterns observed at the mRNA level (Fig. 1b). The expression of *hsa-miR-181a-5p* increased, and the expression of *SBP2* at the mRNA level reduced over time after treatment with 0 (as a control), 1, 5, 10 and 20 ng/ml IL-1β for 12 h (Fig. 1c).

### SBP2 regulated the biosynthesis of three selenoproteins and oxidation resistance in chondrocytes

**Fig. 2:**
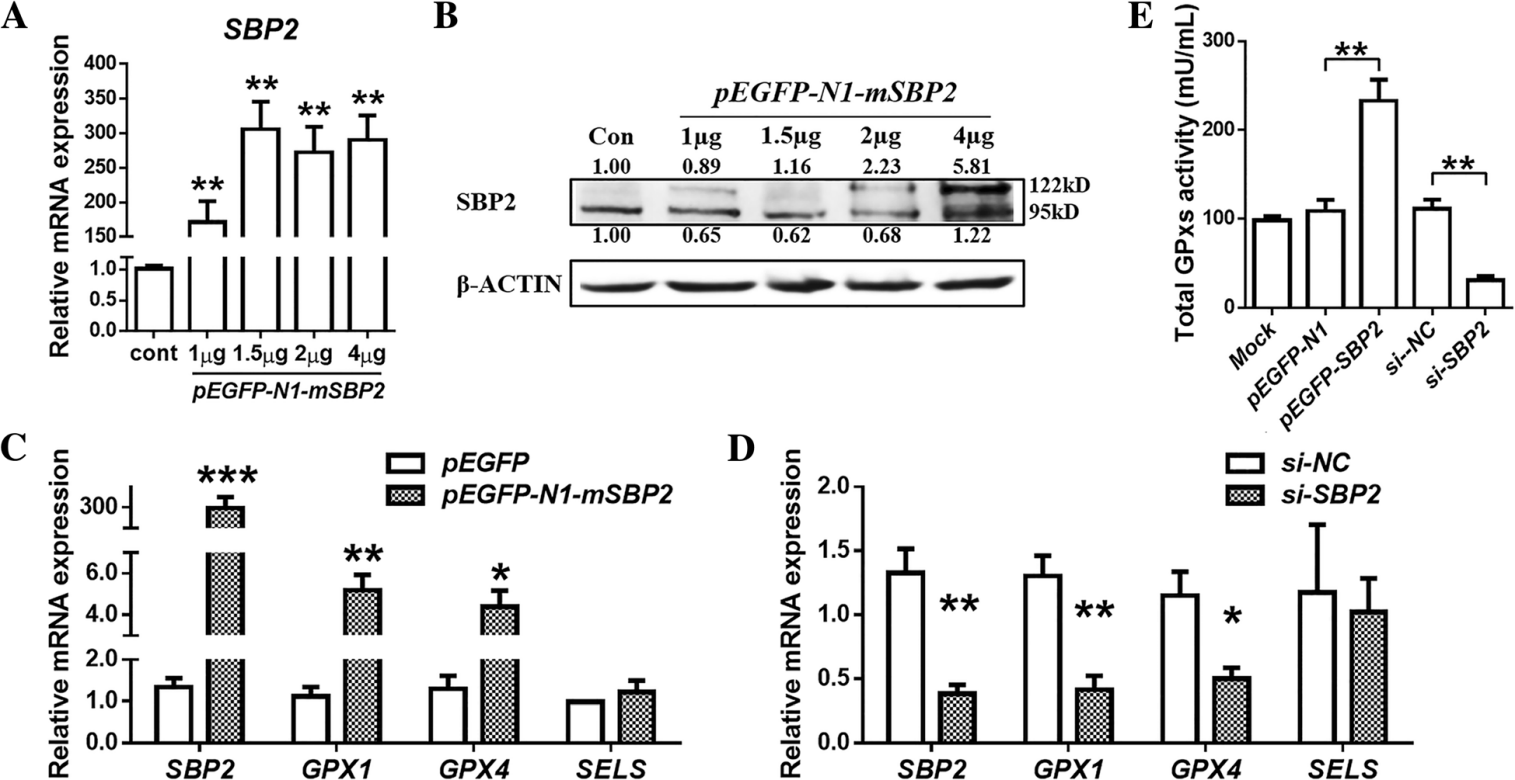
SBP2 regulates the biosynthesis of three selenoproteins and oxidation resistance in chondrocytes. **a** The expression of total (endogenous and exogenous) mSBP2 after transfection with pEGFP-N1-mSBP2 for 24 h in SW1353 cells. (*n* = 3, 3). **b** The expression of endogenous and exogenous SBP2 after transfection with pEGFP-N1-mSBP2 for 24 h in SW1353 cells. **c** The expression of SBP2, GPX1, GPX4 and SELS after transfection with 2 μg of pEGFP-N1-mSBP2 for 24 h in SW1353 cells. (*n* = 3, 3). **d** The expression of SBP2, GPX1, GPX4 and SELS after transfection with si-SBP2 for 24 h in SW1353 cells. (*n* = 3, 3). **e** Total GPXs activity after transfection with pEGFP-N1-mSBP2 or si-SBP2 for 24 h in SW1353 cells. (*n* = 3, 3). The data are expressed as the mean ± SEM; *, ** and *** indicate *P* < 0.05, 0.01 and 0.001, respectively

To assess the role of SBP2 in chondrocytes, we constructed recombinant *hSBP2-CDS* clones and *si-SBP2* (Fig. 2a) and transfected these constructs into SW1353 cells. Exogenous *SBP2* (122 kDa, Fig. 2b) showed remarkable concentration-dependent up-regulation with *pEGFP-N1-mSBP2*. Overall, taking into consideration both endogenous *SBP2* (95 kDa, Fig. 2b) and exogenous *SBP2*, 2 μg of *pEGFP-N1-mSBP2* was the most suitable treatment to achieve *SBP2* over-expression. *SBP2* over-expression (*P =* 0.0003) in SW1353 cells elevated both *GPX1* (*P =* 0.0064) and *GPX4* (*P =* 0.0215) mRNA levels, whereas *SELS* (*P =* 0.4532) induced no evident changes (Fig. 2c). On the other hand, when *SBP2* levels were specifically reduced by *si-SBP2* (*P =* 0.0087), both *GPX1* (*P =* 0.0097) and *GPX4* (*P =* 0.0431) mRNA levels, but not *SELS* levels (*P =* 0.2093), were also down-regulated significantly (Fig. 2d). Meanwhile, total GPXs activity was increased (*P =* 0.0097) by *SBP2* over-expression, and total GPXs activity was reduced (*P =* 0.0023) under *SBP2* knockdown conditions (Fig. 2e).

### Transfection of miR-181a-5p affects chondrocyte phenotype and oxidation resistance through SBP2

**Fig. 3:**
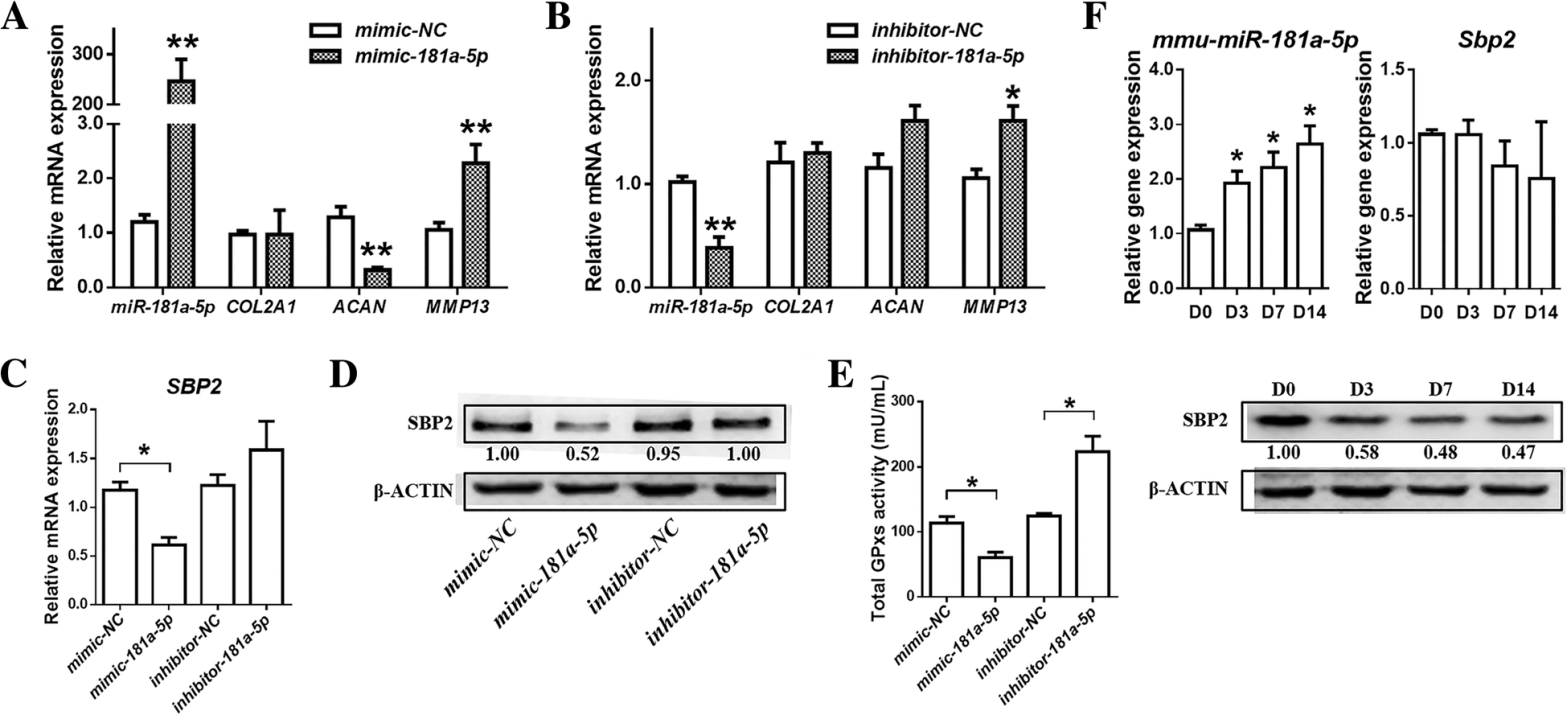
Transfection of miR-181a-5p affects the phenotype and oxidation resistance of chondrocytes through SBP2. **a** The expression of hsa-miR-181a-5p, COL2A1, ACAN and MMP13 after transfection with mimic-181a-5p for 24 h in SW1353 cells. (*n* = 3, 3). **b** The expression of hsa-miR-181a-5p, COL2A1, ACAN and MMP13 after transfection with inhibitor-181a-5p for 24 h in SW1353 cells. (*n* = 3, 3). **c** The expression of SBP2 after transfection with mimic-181a-5p or inhibitor-181a-5p for 24 h in SW1353 cells. (*n* = 3, 3). **d** The expression of SBP2 after transfection with mimic-181a-5p or inhibitor-181a-5p for 24 h in SW1353 cells. **e** Total GPXs activity after transfection with mimic-181a-5p or inhibitor-181a-5p for 24 h in SW1353 cells. (*n* = 3, 3). **f** The expression of mmu-miR-181a-5p, Sbp2 and SBP2 following ITS treatment in ATDC5 cells. (*n* = 3, 3). The data are expressed as the mean ± SEM; *, ** and *** indicate *P* < 0.05, 0.01 and 0.001, respectively

To confirm the roles of *miR-181a-5p* in chondrocytes, a *miR-181a-5p* mimic (*P =* 0.0022) or a *miR-181a-5p* inhibitor (*P =* 0.0108) was applied to alter *miR-181a-5p* levels (Additional file 1: Figure S1). The expression of cartilage-specific genes such as *COL2A1*, *ACAN* and *MMP13* and total GPXs activity were detected in SW1353 cells after transfection for 24 h. First, *mimic-miR-181a-5p* down-regulated *ACAN* (*P =* 0.0052) and up-regulated *MMP13* (*P =* 0.0095) (Fig. 3a), while *inhibitor-miR-181a-5p* also significantly up-regulated *MMP13* (*P =* 0.0319) (Fig. 3b). Furthermore, both *SBP2* (*P* = 0.0209) and SBP2 were significantly down-regulated in SW1353 cells when *miR-181a-5p* was up-regulated by *mimic-181a-5p* (Fig. 3c and d). In contrast, neither *SBP2* nor SBP2 expression changed when *miR-181a-5p* was down-regulated by *inhibitor-181a-5p* (Fig. 3c and d). Meanwhile, total GPXs activity was reduced (*P =* 0.0145) by *miR-181a-5p* over-expression, and total GPXs activity was increased (*P =* 0.0143) under *miR-181a-5p* knockdown conditions (Fig. 3e). In addition, ITS treatment was applied to cultured cells for 14 days as described previously to induce ATDC5 cells to differentiate in vitro [29], and then the expression of *mmu-miR-181a-5p*, *Sbp2* and SBP2 was detected. With chondrocyte differentiation, the expression of *mmu-miR-181a-5p* showed remarkable up-regulation at D3 (*P* = 0.0258), D7 (*P* = 0.0178) and D14 (*P* = 0.0103), while SBP2 protein expression was significantly reduced, although the expression of *Sbp2* was almost constant (Fig. 3f).

### The expression of hsa-miRNA-181a-5p, SBP2 and selenoproteins in OA cartilage

**Fig. 4:**
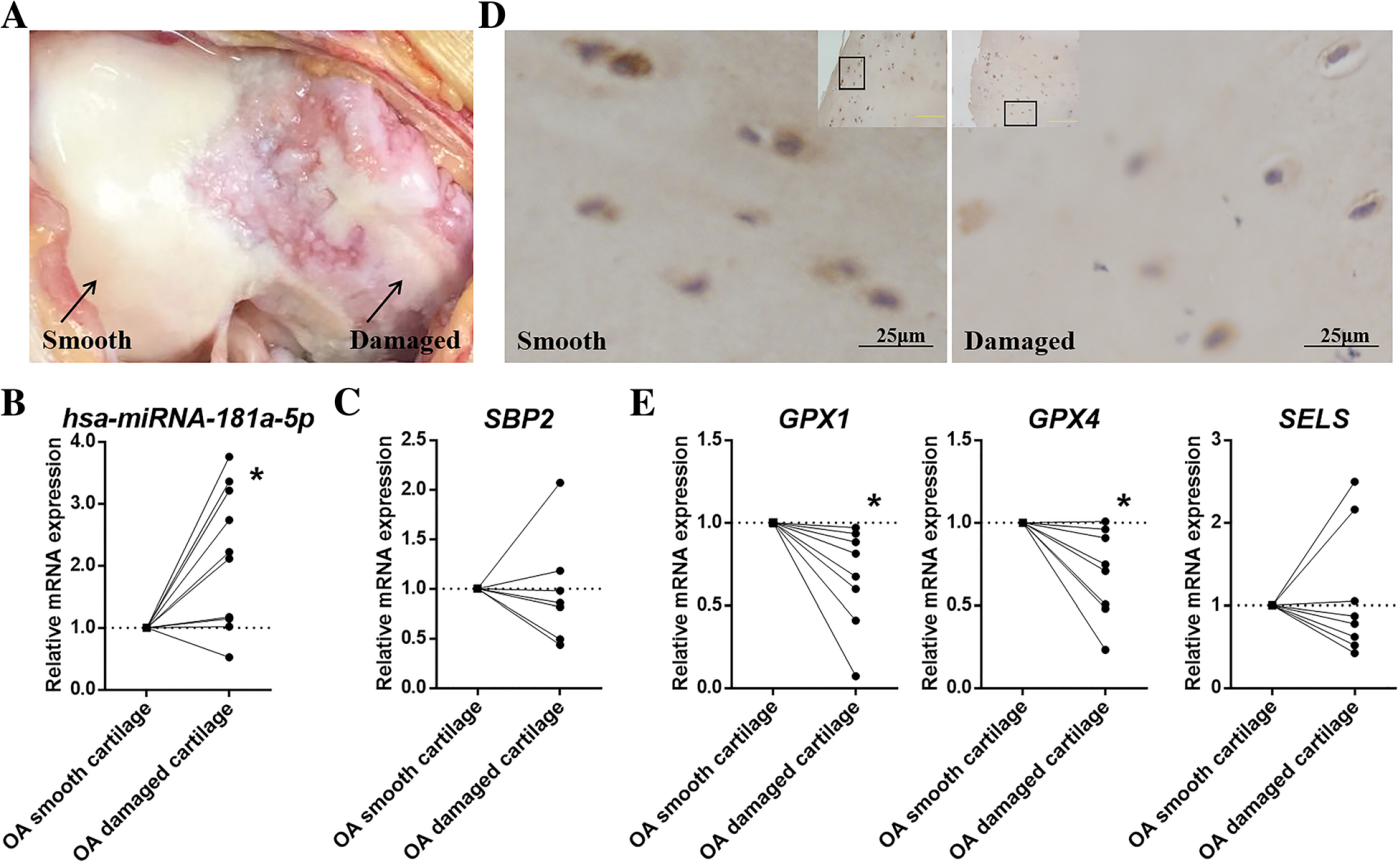
The expression of hsa-miRNA-181a-5p, SBP2 and selenoproteins in OA cartilage. **a** OA smooth cartilage and damaged cartilage from the same patients undergoing total knee replacement. **b** The expression of *hsa-miRNA-181a-5p* in smooth cartilage and damaged cartilage from the same OA cartilage sample. (*n* = 10). **c** The expression of *SBP2* in smooth cartilage and damaged cartilage from the same OA cartilage sample. (*n* = 7). **d** The expression of SBP2 in smooth cartilage and damaged cartilage from the same OA cartilage sample. **e** The expression of *GPX1*, *GPX4* and *SELS* in smooth cartilage and damaged cartilage from the same OA cartilage sample. (*n* = 8). The data were expressed as the mean ± SEM; *, ** and *** indicate *P* < 0.05, 0.01 and 0.001, respectively

Cartilage tissues were obtained from 8 OA patients to detect the expression of *miRNA-181a-5p*, SBP2 and three pivotal selenoproteins. OA smooth cartilage and damaged cartilage from the same patients undergoing TKR were separated (Fig. 4a). Total RNA was extracted, and RT-qPCR was performed. According to a paired Student’s t test, *miRNA-181a-5p* expression levels were significantly higher (*P* = 0.0114) in damaged cartilage than in smooth cartilage of OA patients (Fig. 4b). Meanwhile, although *SBP2* mRNA expression was unattenuated in damaged cartilage (Fig. 4c), SBP2 protein expression was reduced in damaged cartilage (Fig. 4d). Furthermore, *GPX1* (*P* = 0.0183) and *GPX4* (*P* = 0.0149) were down-regulated in damaged OA cartilage (Fig. 4e), while *SELS* showed no significant changes (Fig. 4e).

### The expression of hsa-miRNA-181a-5p, SBP2 and selenoproteins in peripheral blood

**Fig. 5:**
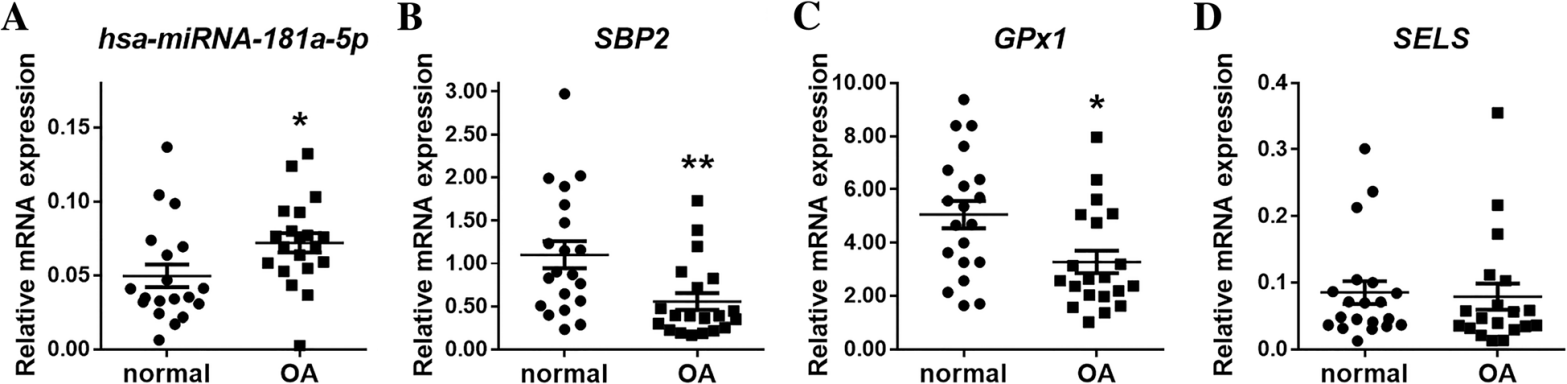
The expression of hsa-miRNA-181a-5p, SBP2 and selenoproteins in peripheral blood. **a** The expression of hsa-miRNA-181a-5p in the peripheral blood of healthy controls and OA patients. (*n* = 19, 20). **b** The expression of SBP2 in the peripheral blood of healthy controls and OA patients. (*n* = 20, 20). **c** The expression of GPX1 in the peripheral blood of healthy controls and OA patients. (*n* = 20, 20). **d** The expression of SELS in the peripheral blood of healthy controls and OA patients. (*n* = 20, 19). The data are expressed as the mean ± SEM; *, ** and *** indicate *P* < 0.05, 0.01 and 0.001, respectively

Peripheral blood was collected from 20 healthy controls and 20 OA patients. To detect the expression of *miRNA-181a-5p*, *SBP2, GPX1*, *GPX4* and *SELS*, total RNA from peripheral blood was extracted, and RT-qPCR was performed. The expression of *hsa-miRNA-181a-5p* (*P* = 0.0329) in OA peripheral blood was significantly higher than that in normal controls (Fig. 5a), while *SBP2* (*P* = 0.0061) and *GPX1* (*P* = 0.0111) were both lower in OA peripheral blood than in normal controls (Fig. 5b and c). In addition, *SELS* (*P* = 0.8160) showed no statistically significant differences (Fig. 5d), and *GPX4* was not detected (data not shown). These results suggested that *hsa-miRNA-181a-5p* is a potential diagnostic biomarker for OA.

## Discussion

To explore whether *miR-181a-5p* and *SBP2* are involved in OA pathogenesis, we established an IL-1β model using the chondrocyte SW1353 cell line. The results showed that IL-1β increased *hsa-miR-181a-5p* and decreased *SBP2* in a time- and dose-dependent manner, while both *hsa-miR-181a-5p* and *SBP2* seemed to participate in the catabolism pathway and oxidative stress in chondrocytes induced by IL-1β. This finding is in line with our expectation that pro-inflammatory cytokines induce *miR-181a-5p* up-regulation in chondrocytes along with SBP2 down-regulation. Coincidentally, *miR-181a-5p* up-regulates the expression of caspase-3, PARP, MMP-2, and MMP-9 while repressing chondrocyte proliferation and promoting chondrocyte apoptosis in OA [22, 30].

Next, we used recombinant plasmids and siRNA sequences targeting *SBP2* to up- or down-regulate the expression of this gene in SW1353 cells. To investigate SBP2-mediated selenoprotein synthesis, *GPX1*, *GPX4* and *SELS* were selected as representative selenoproteins expressed by chondrocytes in this study not only because these proteins exhibit differential cellular localization and fulfil different functions in physiological and pathological processes in various cells but also because the affinity of their SECIS binding with ‘UGA’ recoding has been categorized as strong, moderate and weak, respectively [31, 32]

As crucial antioxidant enzymes*, GPX1* and *GPX4* were regulated by *SBP2* up- or down-regulation, while *SELS* expression levels were always stabilized; these expression patterns are attributable to the differential SECIS affinities and SBP2 binding efficiencies of these proteins. Our findings suggest that *SBP2* expression did not align with selenoprotein expression regulation, which affected total GPXs activity and oxidation resistance in chondrocytes. Oxidative damage due to the concomitant overproduction of ROS is present in ageing and OA cartilage [33]. Predictably, oxidative stress destroys normal physiological signalling and contributes to OA [13]. The synergy between blocked selenoprotein expression and disordered metabolism of the articular cartilage ECM induces chondrocyte apoptosis and contributes to cartilage destruction [9, 34] In summary, selenoprotein biosynthesis leads to decreased antioxidant stress.

Additionally, we modulated *miR-181a-5p* expression by using mimic and inhibitor sequences in SW1353 cells. The expression of *miR-181a-5p* showed remarkable up-regulation, while SBP2 protein expression was significantly reduced. Unexpectedly, SBP2 expression did not change after *miR-181a-5p* knockdown, which implies that a very complex regulatory network and multiple modulators are involved in SBP2 expression. Furthermore, *SBP2* showed a significant negative correlation with *miR-181a-5p* during the induced differentiation of ATDC5 cells. These results suggest that *hsa-miR-181a-5p* affects the chondrocyte phenotype by altering oxidation resistance.

The most effective antioxidants are members of the GPx family, but the mechanisms underlying their effects on OA chondrocytes under oxidative stress are not yet fully understood [9]. Our results established that *miR-181a-5p* regulated total GPXs activity by decreasing the expression of *SBP2* in cartilage, leading to chondrocyte apoptosis and cellular damage induced by ROS. SBP2 is required for protection against ROS-induced cellular damage and increased cell survival [35]. For instance, gene mutations in *SBP2* decreased the expression of several selenoproteins, resulting in a complex multisystem selenoprotein deficiency disorder in humans [36], and lipid peroxidation products mediated by free radicals increased in the blood [37]. Further, miR-34a, miR-146a, SOD2, CAT, GPXs and NRF2 are subjected to H_2_O_2_ stimulus in OA chondrocytes [24]. Meanwhile, miR-9 is a OA-related effects of oxidative stress in chondrocytes through targets SIRT1 [23].

Finally, we discovered that *miRNA-181a-5p* expression was increased, and SBP2 protein and *GPX1* and *GPX4* mRNA expression were reduced in damaged cartilage. These results suggest that *hsa-miRNA-181a-5p, GPX1*, *GPX4* and SBP2 all participate in the OA cartilage damage process to a certain extent. Despite the inadequate number of samples, our peripheral blood data partly support the hypothesis that *miR-181a-5p* is released in plasma and may facilitate early-stage diagnosis of OA because it induces ROS to damage cartilage proteins. Currently, few blood-based tests are used for the detection of early-stage OA.

## Conclusions

We have reported a novel pathway in cartilage. Pro-inflammatory factors mediate *miR-181a-5p* expression, and then *miR-181a-5p* regulates the pivotal selenoproteins GPX1 and GPX4 through its target SBP2, resulting in alterations to the overall activity of GPXs, which are the most important oxidation resistance proteins in cartilage. Oxidation resistance involves a series of antioxidants that overcome ROS-related stress to maintain ECM metabolism balance and protect the essential physiological functions of cartilage.

## Additional file

Additional file 1: **Figure S1.** The illustration of possible pathways about miRNA-181a-5p regulated selenoproteins in chondrocytes. The expression of *has-miR-181a-5p* after transfected *mimic-181a-5p* or *inhibitor-181a-5p* for 24 h in SW1353 cells. (*n* = 3, 3). The data were expressed as means ± SEM, *, ** and *** stand for *P* < 0.05, 0.01 and 0.001 respectively. (TIF 535 kb)

## Abbreviations

ACAN: Aggrecan
COL2: type-II collagen
ECM metabolic: Extracellular matrix
HRP: Horseradish peroxidase
IL-1β: Including interleukin-1
IL-6: Interleukin-6
*inhibitor-181a-5p*: *miR-181a-5p inhibitor*
*mimic-181a-5p*: *miR-181a-5p mimics*
OA: Osteoarthritis
PBS: Phosphate buffered saline
ROS: Reactive oxygen species
Se: Selenium
Sec: Selenocysteine
SECIS: Sec insertion sequence
SECISBP2 or SBP2: Sec insertion sequence binding protein 2
Sel: Selenoprotein
TKR: Total knee replacement
TNF-α: Tumour necrosis factor-α
