# Supplementary figures and images for: The hsa-miR-181a-5p reduces oxidation resistance by controlling SECISBP2 in osteoarthritis
Source: BMC Musculoskelet Disord. 2018 Oct 5;19:355. doi: 10.1186/s12891-018-2273-6 (PMC6172777; doi:10.1186/s12891-018-2273-6)

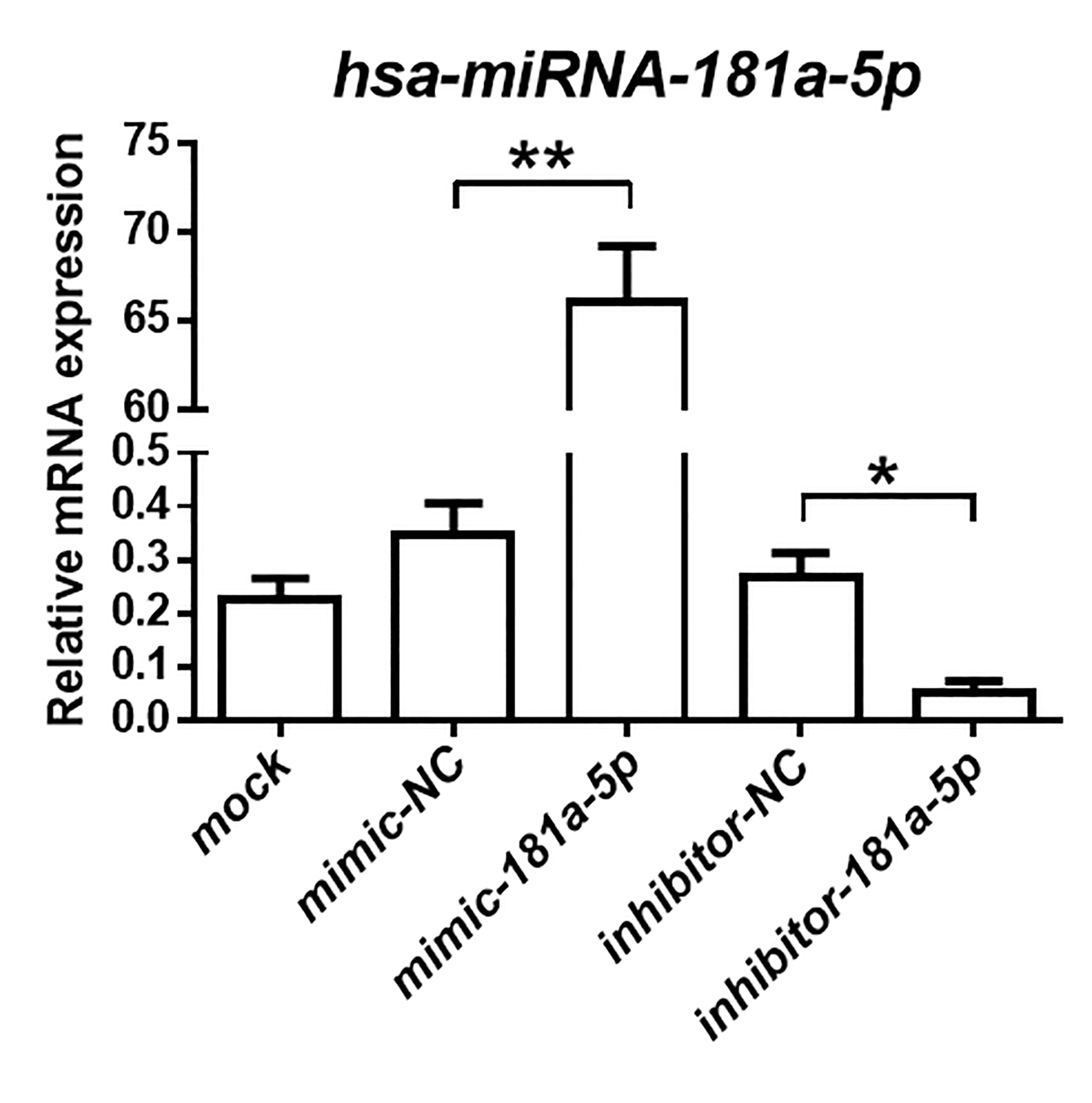

Supplement: Supplementary file 1 — Figure S1. The illustration of possible pathways about miRNA-181a-5p regulated selenoproteins in chondrocytes. The expression of has-miR-181a-5p after transfected mimic-181a-5p or inhibitor-181a-5p for 24 h in SW1353 cells. (n = 3, 3). The data were expressed as means ± SEM, *, ** and *** stand for P < 0.05, 0.01 and 0.001 respectively. (TIF 535 kb) [file 12891_2018_2273_MOESM1_ESM.tif]
